# Body Mass Index at Accession and Incident Cardiometabolic Risk Factors in US Army Soldiers, 2001–2011

**DOI:** 10.1371/journal.pone.0170144

**Published:** 2017-01-17

**Authors:** Adela Hruby, Lakmini Bulathsinhala, Craig J. McKinnon, Owen T. Hill, Scott J. Montain, Andrew J. Young, Tracey J. Smith

**Affiliations:** 1 Military Nutrition Division, US Army Research Institute of Environmental Medicine, Natick, Massachusetts, United States of America; 2 Nutritional Epidemiology Program, Jean Mayer USDA Human Nutrition Research Center on Aging at Tufts University, Boston, Massachusetts, United States of America; 3 Military Performance Division, US Army Research Institute of Environmental Medicine, Natick, Massachusetts, United States of America; 4 U.S. Army Medical Department Center and School, Health Readiness Center of Excellence, Fort Sam, Houston, TX, United States of America; Dasman Diabetes Institute, KUWAIT

## Abstract

Individuals entering US Army service are generally young and healthy, but many are overweight, which may impact cardiometabolic risk despite physical activity and fitness requirements. This analysis examines the association between Soldiers’ BMI at accession and incident cardiometabolic risk factors (CRF) using longitudinal data from 731,014 Soldiers (17.0% female; age: 21.6 [3.9] years; BMI: 24.7 [3.8] kg/m^2^) who were assessed at Army accession, 2001–2011. CRF were defined as incident diagnoses through 2011, by ICD-9 code, of metabolic syndrome, glucose/insulin disorder, hypertension, dyslipidemia, or overweight/obesity (in those not initially overweight/obese). Multivariable-adjusted proportional hazards models were used to estimate hazard ratios (HR) and 95% confidence intervals (CI) between BMI categories at accession and CRF. Initially underweight (BMI<18.5 kg/m^2^) were 2.4% of Soldiers, 53.5% were normal weight (18.5−<25), 34.2% were overweight (25−<30), and 10.0% were obese (≥30). Mean age range at CRF diagnosis was 24–29 years old, with generally low CRF incidence: 228 with metabolic syndrome, 3,880 with a glucose/insulin disorder, 26,373 with hypertension, and 13,404 with dyslipidemia. Of the Soldiers who were not overweight or obese at accession, 5,361 were eventually diagnosed as overweight or obese. Relative to Soldiers who were normal weight at accession, those who were overweight or obese, respectively, had significantly higher risk of developing each CRF after multivariable adjustment (HR [95% CI]: metabolic syndrome: 4.13 [2.87–5.94], 13.36 [9.00–19.83]; glucose/insulin disorder: 1.39 [1.30–1.50], 2.76 [2.52–3.04]; hypertension: 1.85 [1.80–1.90], 3.31 [3.20–3.42]; dyslipidemia: 1.81 [1.75–1.89], 3.19 [3.04–3.35]). Risk of hypertension, dyslipidemia, and overweight/obesity in initially underweight Soldiers was 40%, 31%, and 79% lower, respectively, versus normal-weight Soldiers. BMI in early adulthood has important implications for cardiometabolic health, even within young, physically active populations.

## Introduction

Obesity is a well-recognized global health burden. Overweight and obesity increase risk of cardiometabolic diseases and related risk factors, including hypertension, dyslipidemia, disorders of glucose and insulin metabolism including type 2 diabetes (T2D), and heart disease.[1–3] Prospective members of the US Army are drawn from an increasingly overweight/obese civilian population.[4] However, individuals entering (known as “accessing” into) the Army must meet age- and sex-specific weight-for-height screening criteria defined in Army Regulation 40–501: *Standards of Medical Fitness*.[5] When an individual exceeds the maximum allowable weight-for-height criteria, body fat standards, in use since 1991, are the final determinant in evaluating an applicant’s acceptability.[5] While the weight-for-height criteria generally align with body mass index (BMI)-based classifications[6] of underweight through obesity, they allow for individuals to have a BMI in the overweight range (25–29.9 kg/m^2^).[7,8]

Soldiers are also required to meet body fat[9] and fitness standards[5], and to engage in physical training[10,11] during service. Regular physical activity and/or fitness are associated with reduced risk of cardiometabolic disease, and may at least partially offset risk associated with overweight/obesity.[12,13] Despite the existing fitness and training requirements of Soldiers, cardiometabolic disease is nevertheless prevalent, with prior studies highlighting risk factors present in new and active-duty military personnel. For example, in 209 new recruits entering Basic Combat Training in 2010, Pasiakos and colleagues[14] reported >10% had obesity, dyslipidemia, or hyperglycemia. In a cross-sectional study of 659 Soldiers who were in a mandatory weight-management class (mean age 29 years, 69% obese), 30% had high cholesterol, 70% had high low-density-lipoprotein (LDL) cholesterol, 49% had low high-density lipoprotein (HDL) cholesterol, and 29% had high triglycerides.[15] Autopsy data from Soldiers who had died of combat or unintentional injuries (mean age 26 years) between 2001 and 2011 indicated that 8.5% had coronary atherosclerosis and 2.3% had severe coronary atherosclerosis; furthermore, those with medical-record evidence of dyslipidemia, hypertension, and/or obesity had a higher prevalence of atherosclerosis.[16]

Excess weight results in lost work days due to illness or injury, increases in medical costs, reduced quality of life, and earlier departure from service. In a 2002–2006 study of over 265,000 Soldiers examining attrition in the first year of service, those with a BMI >34 kg/m^2^ had 47% higher odds of all-cause and 68% higher odds of medical discharge during the first year of service, compared with those with a BMI of 24–24.9 kg/m^2^.[17] This trend is borne out in the longer-term as well: an analysis of 1998–2010 military medical surveillance data found that Soldiers with overweight-related diagnoses left service a median of 1.21 years before matched controls.[18]

Existing data thus suggest that Soldiers, despite mandatory fitness requirements, are not immune to the health and professional sequelae of excess weight. Furthermore, risks associated with excess weight prior to enlisting may be an important modifiable risk factor meriting greater attention both before and while in service. The purpose of this study is to examine how weight status upon entering (i.e., accession into) the US Army impacts the long-term health of Soldiers with respect to incident cardiometabolic risk factor (CRF) diagnoses. Studies in certain groups of Soldiers indicate they are highly physically fit[19–22], and all Soldiers are required to participate in mandatory physical training, meet fitness standards, and adhere to a physical readiness ethos.[10,11] Soldier fitness may be protective against CRF, despite highly prevalent excess weight at accession.[4] Our primary hypothesis, however, is that, as in civilians[23–26], even within this generally young population that regularly engages in physical activity, overweight and obesity at accession increase risk of incident CRF later during a Soldier’s career. If this hypothesis is supported, there may be need for ongoing review of current screening criteria, higher levels of intervention before overweight/obese individuals are allowed to access into the Army, and/or a bolstering of support for existing Army weight-management programs.

## Methods

### Study design and participants

This study, conducted January–April 2016 with approval from the Institutional Review Board, US Army Research Institute of Environmental Medicine (Natick, MA), used existing historical data from the Total Army Injury and Health Outcomes Database (TAIHOD) for the years 2001–2011. Consent was not required, since data was analyzed anonymously. The TAIHOD is a data repository that maintains records of administrative and health-related datasets on Active Duty Soldiers, to support epidemiologic research.[27] Data from the TAIHOD on enlisted personnel (excluding commissioned officers) were drawn from the following datasets: Military Entrance Processing Command dataset, 2001–2011: date, height, weight, and International Classification of Diseases (ICD-9) codes of CRFs at application; Defense Manpower Data Center Master Personnel and Transaction dataset, 2001–2011: date of birth, sex, race/ethnicity, education, and marital status; and ICD-9 codes and dates corresponding to CRFs from inpatient/outpatient records from four clinical encounter datasets, 2002–2011 (Standard Inpatient Data Record: military medical treatment facility and civilian hospital admissions; Standard Ambulatory Data Record and Comprehensive Ambulatory Provider Encounter Record: outpatient visits; and TRICARE Encounter Data—Institutional and Non-Institutional). The TAIHOD administrators entered into data use agreements with Defense Manpower Data Center (DMDC) and the Defense Health Agency (DHA) to obtain personnel and medical encounter data, respectively. The medical encounter data is from the Military Health System Data Repository (MDR). Authors cannot legally share the dataset due to legal restrictions within the data use agreements; however, interested parties may seek to establish data use agreements by contacting the Military Health System Data Repository (MDR) and the Defense Manpower Data Center (DMDC) via the following websites, respectively, http://health.mil/Military-Health-Topics/Privacy-and-Civil-Liberties/Submit-a-Data-Sharing-Application, and http://www.dmdc.osd.mil/appj/dwp/data_reqs.jsp. The interval between application and successful accession into the Army can be up to 18 months.[28] There were 738,046 unique observations indicating individuals successfully accessed into the Army for the first time at some point between 1 January 2001 and 31 December 2011, and were thereafter considered Army personnel, and thus eligible for follow-up until an outcome occurred, separation from the Army, or study cutoff (31 December 2011). Excluded were 1,988 (0.27%) participants missing or having implausible recorded height (<1.37 or ≥2.13 m), weight (<36.3 or ≥204.1 kg) or calculated BMI (<10 or ≥50 kg/m^2^); 613 (0.08%) missing information on date of birth, 2 missing information on sex, and 4,429 (0.60%) with any baseline CRF (except overweight/obesity) recorded at accession, for a total of 731,014 participants included in the analysis.

### Body size measures and categories

Participants were categorized by baseline BMI (kg/m^2^) according to national guidelines as underweight (<18.5 kg/m^2^), normal weight (≥18.5–<25 kg/m^2^), overweight (≥25–<30 kg/m^2^), or obese (≥30 kg/m^2^).[6] Screening Table Weights (STW) from Army Regulation 40–501 (*Standards of Medical Fitness*) were used to categorize participants as under, meeting, or exceeding the weight-for-height criteria for accession in a given year.[5] Men and women entering the Army must meet age- and sex-specific weight-for-height screening criteria defined in Army Regulation 40–501, which are different from BMI thresholds[6] and were developed to align with a range of healthy BMI and body fat.[7,8] When an individual exceeds the maximum allowable weight-for-height criteria, body fat standards, in use since 1991, are the final determinant in evaluating an applicant’s acceptability.[5] These screening criteria and body fat standards have changed over time, as described for the time period of interest in **S1** and **S2 Tables** in the **Supporting Information**. In analyses, the criteria in use at the time of a given Soldier’s accession were applied.

### Outcome definitions

Primary outcomes were any post-accession diagnosis of a CRF, including hypertension, disorder of glucose or insulin metabolism (e.g., insulin use, type 2 diabetes), dyslipidemia (e.g., low HDL cholesterol, high triglycerides), and metabolic syndrome, as well as overweight/obesity (among initially underweight and normal-weight Soldiers), as identified via ICD-9 diagnostic codes. We developed both broad (primary analysis) and strict (secondary analysis) ICD-9-based definitions of these conditions, as listed in **S3 Table**. Participants were considered cases if an incident risk factor diagnostic code appeared in their medical records following their accession date and prior to the last day of the last month in which he/she appeared in personnel files (date of separation from the Army) or the study cutoff, 31 December 2011. The date of diagnosis was used as the event date.

### Covariate ascertainment

Demographics from personnel files included age, sex, educational attainment, race/ethnicity, and marital status. Individuals with missing data on variables other than age, sex, and anthropometry were classified as “Other/Unknown”. Time-varying covariates included primary military occupation and number of overseas deployments in the time period of interest. Specific behavioral (i.e., tobacco and/or alcohol use) and other risk factors (i.e., depression, anxiety, posttraumatic stress disorder) were defined by ICD-9 code (S3 Table), if they occurred prior to the date of diagnosis of cardiometabolic risk factor(s) for cases, or the censoring date (date of separation from the Army) or the study cutoff, 31 December 2011) for non-cases.

### Statistical analysis

Unadjusted percentages and means (standard deviations [SD]) of participant characteristics at baseline (accession) are presented by BMI category. Each Soldier’s person-time (in months) at risk was calculated from the date of accession to the date of each (incident) CRF, date of separation from the Army, or study cutoff, whichever occurred first. To estimate the risk of each incident CRF as a function of BMI category, crude incidence, incidence rates, were calculated, and hazard ratios (HR) and 95% confidence intervals (CI) were estimated using Cox proportional hazards models with normal BMI (18.5–<25 kg/m^2^) as the reference. Two models were considered: model 1 was adjusted for baseline age and sex; model 2 was additionally adjusted for race/ethnicity, educational attainment, and marital status. Additional adjustment model 2 for time-varying primary military occupation and deployment in the follow-up period was also considered, as was adjustment for behavioral risk factors and mental health/addiction disorders. In a secondary model, we explored the potentially mediating role of incident diagnoses of overweight/obesity in subsequent incident CRFs by adjusting for an overweight/obesity diagnosis if it preceded a CRF of interest. To assess if year of accession affected risk estimates, we also generated models stratified by year of accession. Survival curves were inspected for deviation from proportional hazards assumptions. In sensitivity analyses, to examine potential bias created by those who accessed into the Army between 2009 and 2011 (potentially artificially cutting off follow-up time), the primary analysis was repeated with only those who accessed before 2009. Potential effect modification by sex was considered by stratifying by sex and comparing results between sexes. The primary regressions described above were repeated using STW categories (under, meeting, over STW) in place of BMI categories, using “meeting STW” as the reference.

Finally, we conducted exploratory analyses in those diagnosed with one or more CRFs to better understand the onset and sequence of CRFs: we explored the number of diagnoses, time to and age at diagnoses, as well as the most common order of appearance of diagnoses in each BMI category. To iterate previous reports of attrition in the first year of service[17] and shorter service durations[18] in overweight/obese Soldiers, we also explored whether BMI at accession was associated with service duration (defined as time from the date of accession to the date of Army separation or the study cutoff, whichever occurred first) in those Soldiers who were not diagnosed with any incident CRF.

Statistical procedures were performed using SAS (v9.3, SAS Institute, Cary, NC). While a two-sided alpha <0.05 was considered statistically significant, owing to the very large sample size, *P* values for many statistical tests were <0.001. Therefore, point estimates and confidence intervals are preferred to *P* values as indicators of strength and consistency of associations.

## Results

Baseline characteristics of participants by BMI category and the total population are presented in **Table 1**. On average, Soldiers included in the analysis were 21.6 (3.9) years old when they accessed into the Army, 17% female, 2.4% underweight, 53.5% normal weight, 34.2% overweight, and 10.0% obese.

**Table 1:** Baseline characteristics by BMI category of 731,014 men and women accessing into the US Army, 2001–2011.

	Category of BMI (kg/m^2^)				
	Underweight	Normal weight	Overweight	Obese	
	<18.5	18.5 to <25	25 to <30	≥30	Total
***N***	17,478	390,738	249,823	72,975	731,014
**Female, %**	27.46	21.06	14.32	2.26	17.04
**Age, years ^a^**	20.79 (3.37)	21.22 (3.69)	22.22 (4.19)	22.34 (4.03)	21.66 (3.93)
**BMI, kg/m^2^^a^**	17.91 (0.55)	22.15 (1.72)	27.14 (1.38)	32.07 (1.77)	24.74 (3.82)
**Age category, %**					
< 20 years	56.21	51.30	39.30	35.17	45.71
20 to < 30 years	40.70	44.70	54.21	58.64	49.25
30 to < 40 years	3.04	3.88	6.30	6.06	4.91
≥ 40 years	0.05	0.12	0.18	0.13	0.14
**Meets STW, %**					
Under STW	41.62	0.44	0.00	0.00	1.23
Meets STW	58.26	96.56	50.42	0.00	70.24
Above STW	0.12	3.00	49.58	100.00	28.53
**Race/Ethnicity, %**					
White	67.02	66.82	66.78	63.12	66.44
Black	17.95	17.59	14.66	17.55	16.60
Hispanic	9.47	10.27	13.15	14.06	11.61
Asian/Pacific Islander	4.39	4.07	4.07	4.1	4.08
Indian/Alaskan	0.83	1.01	1.1	0.97	1.03
Other/Unknown	0.33	0.24	0.23	0.18	0.24
**Education, %**					
<High school	3.56	3.6	4	3.56	3.73
High school	63.58	63.96	62.88	64.65		63.65
College/Some college	19.96	19.55	20.78	19.34	19.96
Advanced degree	0.10	0.28	0.44	0.27	0.33
Other/Unknown	12.81	12.61	11.91	12.18	12.33
**Marital status, %**					
Never married	84.7	84.06	77.82	76.15	81.15
Married	14.23	14.39	20.08	21.98	17.09
Divorced/Separated/ Widowed	1.03	1.48	2.02	1.8	1.68
Other/Unknown	0.04	0.07	0.08	0.07	0.07
**Occupation, %**					
Infantry/Gun Crews	43.31	48.16	51.11	52.19	49.46
Electr. Equip. Repair	5.42	4.93	4.67	4.92	4.85
Communications/Intel.	8.63	8.29	7.99	8.45	8.21
Enlisted Health Care	5.13	5.35	5.28	4.69	5.26
Technic/Allied Special	2.44	2.39	2.27	2.12	2.32
Support/Admin.	10.84	8.25	6.33	4.43	7.27
Elect./Mechan. Equip Rep	10.20	9.03	8.55	8.99	8.89
Craftsworkers	1.54	1.51	1.45	1.41	1.48
Service/Supply	10.65	9.63	9.35	10.40	9.64
Non-occupational Enlisted	1.85	2.45	3.00	2.41	2.62
Other	0.00	0.01	0.01	0.00	0.01
**Number of deployments ^a^^,^^b^**	1.61 (0.94)	1.64 (1.01)	1.60 (0.98)	1.52 (0.86)	1.61 (0.99)

Abbreviations: BMI, body mass index; STW, standard table weight.

^**a**^Mean (SD).

^**b**^In the follow-up period.

A higher proportion of men than women were overweight (35.3 vs. 28.7%, respectively) or obese (11.8 vs. 1.3%, respectively). Obesity prevalence was highest among 20–30 year and 30–40 year age groups (11.9 and 12.3%, respectively), with the highest proportion of overweight in those 40+ years at accession (44.4%). Soldiers of Hispanic ethnicity tended to have the highest proportion of overweight (38.7%) and obesity (12.1%), followed by Blacks (10.6% obese and 30.2% overweight), Asian/Pacific Islanders (10.0% obese and 34.1% overweight), and Whites (9.5% obese and 34.4% overweight). Married Soldiers had the highest obesity prevalence (12.8%), while those who were divorced, separated, or widowed had the highest overweight prevalence (41.0%).

### Risk of incident CRF

Across a mean follow-up time of 3.2 years (median 2.9 years), we observed 228 cases of metabolic syndrome (by single ICD-9 code), 3,880 cases of impaired glucose/insulin disorder, 26,373 cases of hypertension, and 13,404 cases of dyslipidemia, and 5,361 cases of overweight/obesity among those with a BMI initially <25 kg/m^2^. Overall, 5.69% (*N* = 41,582) of Soldiers had at least one diagnosed CRF. Compared with Soldiers who accessed at a normal weight, overweight/obesity at accession incrementally raised risk of being diagnosed with a given CRF (**Table 2**).

**Table 2:** Hazard ratios (95% confidence intervals) of broadly defined cardiometabolic risks across BMI categories at accession among 731,014 US Army entrants, 2001–2011.

			**BMI Category (kg/m**^**2**^**)**				
Outcome	Model^a^	Total Events	Underweight (<18.5)	Normal weight (18.5–<25)	Overweight (25–<30)	Obese (≥30)	Per kg/m^2^
Metabolic syndrome (single ICD-9 code)	*Events*	228	2	41	105	80	** **
** **	*Follow-up Time*^b^	** **	690,411	16,038,189	9,970,038	2,782,406	** **
** **	*Crude Rate*^b^	** **	0.0003	0.0003	0.001	0.003	** **
	Model 1		1.10 (0.27–4.55)	1 *(ref*.*)*	4.19 (2.91–6.03)	13.51 (9.12–20.01)	1.29 (1.25–1.33)
	Model 2		1.10 (0.27–4.53)	1 *(ref*.*)*	4.13 (2.87–5.94)	13.36 (9.00–19.83)	1.29 (1.25–1.33)
	Model 3		1.15 (0.28–4.75)	1 *(ref*.*)*	3.68 (2.55–5.30)	10.85 (7.29–16.15)	1.26 (1.22–1.31)
Overweight/ Obesity^c^	*Events*	5,361	54	5 307	--	--	
	*Follow-up Time*^b^		688,552	15,882,406	--	--	
	*Crude Rate*^b^		0.008	0.033	--	--	
	Model 1		0.20 (0.16–0.27)	1 *(ref*.*)*	--	--	1.58 (1.55–1.61)
	Model 2		0.21 (0.16–0.27)	1 *(ref*.*)*	--	--	1.58 (1.55–1.60)
	Model 3		0.23 (0.18–0.30)	1 *(ref*.*)*	--	--	1.51 (1.48–1.54)
Impaired glucose/ insulin disorder	*Events*	3,880	75	1,691	1,439	675	
	*Follow-up Time*^b^		688,563	16,001,619	9,944,631	2,772,559	
	*Crude Rate*^b^		0.011	0.011	0.014	0.024	
	Model 1		0.98 (0.78–1.24)	1 *(ref*.*)*	1.41 (1.31–1.51)	2.85 (2.59–3.12)	1.10 (1.09–1.11)
	Model 2		0.98 (0.78–1.23)	1 *(ref*.*)*	1.39 (1.30–1.50)	2.76 (2.52–3.04)	1.10 (1.09–1.11)
	Model 3		1.05 (0.84–1.33)	1 *(ref*.*)*	1.18 (1.09–1.26)	2.05 (1.86–2.25)	1.07 (1.06–1.07)
Hypertension	*Events*	26,373	241	9,455	11,195	5,482	
	*Follow-up Time*^b^		684,149	15,808,503	9,712,588	2,663,809	
	*Crude Rate*^b^		0.035	0.060	0.115	0.206	
	Model 1		0.60 (0.53–0.69)	1 *(ref*.*)*	1.82 (1.77–1.87)	3.35 (3.24–3.47)	1.13 (1.13–1.14)
	Model 2		0.60 (0.53–0.69)	1 *(ref*.*)*	1.85 (1.80–1.90)	3.31 (3.20–3.42)	1.13 (1.13–1.13)
	Model 3		0.63 (0.56–0.72)	1 *(ref*.*)*	1.59 (1.54–1.63)	2.44 (2.36–2.53)	1.10 (1.09–1.10)
Dyslipidemia	*Events*	13,404	132	4,685	5,855	2,732	
	*Follow-up Time*^b^		687,485	15,936,094	9,847,079	2,730,696	
	*Crude Rate*^b^		0.019	0.029	0.059	0.100	
	Model 1		0.68 (0.58–0.81)	1 *(ref*.*)*	1.85 (1.78–1.92)	3.24 (3.09–3.40)	1.13 (1.12–1.13)
	Model 2		0.69 (0.58–0.81)	1 *(ref*.*)*	1.81 (1.75–1.89)	3.19 (3.04–3.35)	1.13 (1.12–1.13)
	Model 3		0.72 (0.60–0.85)	1 *(ref*.*)*	1.56 (1.50–1.62)	2.36 (2.24–2.48)	1.09 (1.09–1.10)

Abbreviations: BMI, body mass index; ICD, International Classification of Diseases.

^**a**^Model adjustments as follows: Model 1 was adjusted for age at baseline (<20, 20–<30, 30–<40, 40+ years) and sex. Model 2 was adjusted as for Model 1, plus the following demographic covariates: race/ethnicity (white, black, Hispanic, Asian/Pacific Islander, Indian/Alaskan, other/unknown), educational attainment (<high school, some college/college, advanced degree, other/unknown), and marital status (never married, married, divorced/separated/widowed, other/unknown). Model 3 was adjusted as for Model 2, plus ICD-9 coding for behavioral risk factors (tobacco use, alcohol use) and other risk factors (depression, anxiety, posttraumatic stress disorder) reported prior to the outcome diagnosis.

^**b**^Expressed as/in 100 person-months.

^**c**^Among those with body mass index <25 kg/m^2^ at baseline/accession, *N* = 408,216.

For example, in model 2, risk of incident hypertension was 1.85 times and 3.31 times the risk in normal-weight Soldiers, in those who were overweight or obese at accession, respectively. Soldiers who were underweight at accession had lower risk of most incident CRFs compared to normal-weight Soldiers, except metabolic syndrome and impaired glucose/insulin disorder in which there were no statistically significant differences with normal-weight Soldiers. There were no substantive changes to the results after further adjustment of model 2 for behavioral risk factors or mental health/addiction disorders (model 3), or for occupation or deployment history, nor after stratifying by accession year (data not shown). There was no evidence of effect modification by sex (data not shown). In secondary models adjusting for an incident overweight/obesity diagnosis that preceded another CRF diagnosis, risk estimates were attenuated, but substantively unchanged (data not shown). When outcome definitions were strict (i.e., narrower set of possible ICD-9 codes, see S3 Table), hazard ratios were consistent, but tended to be stronger than for broad outcome definitions (**S4 Table**). Risk estimates from sensitivity analyses limited to those who accessed before 2009 were not materially different than when the full sample was used (data not shown). When STW criteria replaced BMI categories, results were consistent with BMI results; those who exceeded STW were at higher risk, and those who were under STW tended to have lower risk of each CRF than those who met STW (**S5 Table**).

### Exploratory analyses of timing and order of diagnoses

In Soldiers without an incident CRF, age-adjusted mean follow-up time in normal-weight Soldiers was 39.8 months. The adjusted mean follow-up time was 6.2 months shorter in the obese, 2.7 months shorter in the overweight, and 1 month shorter in the underweight.

Overall, 5.69% (*N* = 41 582) of Soldiers had at least one CRF diagnosis. Soldiers with a BMI ≥25 kg/m^2^ at accession could have had up to four incident CRFs, while those with a BMI <25 kg/m^2^ at accession could have had up to five incident CRFs (i.e., including incident overweight/obesity). Of the 322,798 initially overweight/obese Soldiers, 7.09% had one or more incident CRFs (6.28% of overweight and 8.80% of obese); and just 23 of these Soldiers had all four possible CRFs, 563 had three, 3,468 had two, and 18,846 (5.84%) had one. Of 408,216 initially underweight/normal-weight Soldiers, 4.58% had one or more incident CRFs (2.55% of underweight and 4.67% of normal-weight); and just 2 of these Soldiers had all five possible CRFs, 38 had four, 360 had three, 2,159 had two, and 16,123 (3.95%) had one.

In those with an incident CRF, the order of CRF diagnoses, adjusted for age at accession, sex, and accession year, remained largely consistent across BMI categories: overweight/obesity came first (in those initially underweight or normal-weight), followed by hypertension, impaired glucose/insulin disorder, metabolic syndrome, and dyslipidemia (**Table 3**).

**Table 3:** Adjusted mean time ± standard error to diagnosis (in months from accession) of broadly defined cardiometabolic risks across BMI categories at accession among 731 014 Army entrants, 2001–2011.^a^

Body Mass Index Category (kg/m^2^)	Hypertension	Dyslipidemia	Glucose/insulin disorder	Metabolic syndrome	Overweight/ Obesity^b^
Underweight (<18.5)	39.51 ± 1.54	49.71 ±2.14	36.45 ± 3.01	54.21 ± 17.00	35.19 ± 3.04
Normal weight (18.5–<25)	40.60 ± 0.25	50.76 ±0.36	41.44 ± 0.64	57.77 ± 3.86	39.00 ± 0.32
Overweight (25–<30)	39.48 ±0.23^c^	48.26 ±0.32^c^	44.00 ± 0.69^d^	49.50 ± 2.34	33.50 ± 0.17^c^
Obese (≥30)	36.42 ±0.33^c^	44.76 ±0.48^c^	44.22 ±1.03^d^	46.19 ± 2.76^c^	29.16 ± 0.22^c^

^a^Time to diagnosis is expressed as mean adjusted time in months (standard error). Means were adjusted for age at accession, sex, and year of accession.

^b^Truly “incident” only in initially underweight or normal weight Soldiers. Time to diagnosis in overweight and obese Soldiers is given for illustrative purposes only.

^c^Significantly different from normal weight, P <0.001.

^d^Significantly different from normal weight, 0.001< P <0.05.

**Fig 1:**
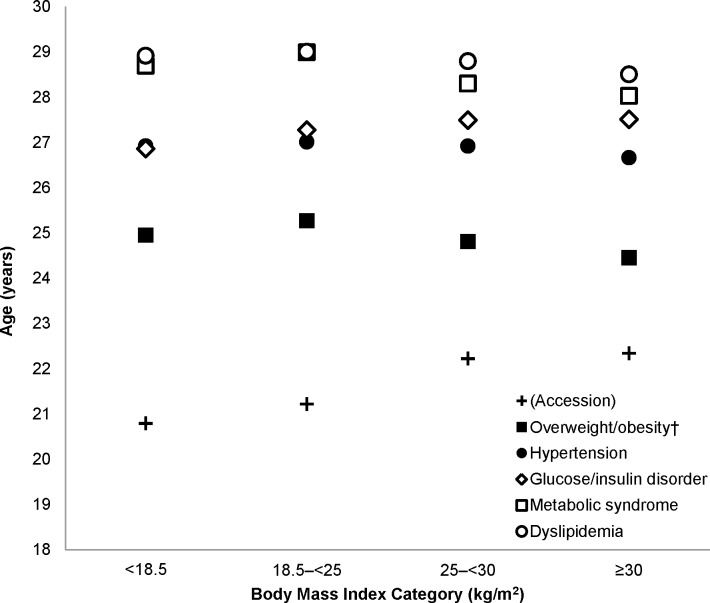
Adjusted mean age (years) at diagnosis of broadly defined cardiometabolic risk factors across body mass index categories at accession among 731,014 US Army Entrants, 2001–2011. Mean ages of incident overweight/obesity (closed squares), hypertension (closed circles), glucose/insulin disorder (open diamonds), metabolic syndrome (open squares), and dyslipidemia (open circles) were adjusted for sex, age at accession, and year of accession. Age at accession is provided for context. †Overweight/obesity is truly “incident” only in initially underweight or normal-weight Soldiers, and these diagnoses in overweight and obese Soldiers is given for context only.

Only in the initially underweight did hypertension slightly follow, as opposed to precede, impaired glucose/insulin disorder. In addition, with the exception of glucose/insulin disorder, each CRF appeared on average significantly earlier in those with BMI ≥25 kg/m^2^ at accession compared with those who were normal weight. Adjusted age at diagnoses (**Fig 1**) followed the time-to-diagnoses trends.

We did not consider “incident” diagnoses of overweight/obesity in those who were overweight/obese at accession, since they may not have been truly incident (i.e., these individuals may or may not have lost weight through Basic Combat Training and/or during their time in service, and the overweight/obesity diagnostic codes may not have been reliably used). However, for exploratory analyses and to provide additional context, we observed that these diagnostic codes appeared with greater frequency in those who were overweight/obese at accession: 7.19% of initially overweight and 15.95% of initially obese had “incident” overweight/obesity diagnostic codes in their medical records, compared with 1.36% of initially normal-weight and 0.31% of initially underweight Soldiers. In addition, as in those with BMI <25 kg/m^2^, these diagnoses in those with BMI ≥25 kg/m^2^ tended to precede other CRF diagnoses.

## Discussion

In the present analysis of over 731,000 Soldiers who accessed into the US Army in 2001−2011, we observed generally low incidence of cardiometabolic risk factor diagnoses. Nevertheless, overweight and obesity conferred substantially increased risks of these conditions even within this young population that regularly engages in physically activity and must meet biannual physical fitness standards. In addition to their youth, the ostensibly high cardiorespiratory fitness in at least some subgroups[19–22] of this population may be one reason why the overall number of incident diagnoses was low, since fitness levels have been shown to modify cardiometabolic risk even in the presence of excess body weight,[29–34] and may be one of the distinguishing characteristics of the “metabolically healthy obese” vs. “metabolically unhealthy obese” phenotypes.[13,35]

Our observations are consistent with research in civilian populations, which has similarly reported higher risk of cardiometabolic morbidity at higher body weight.[23–26,31] Hypertension was the most common incident diagnosis, affecting 3.6% of the present study population, and was the earliest diagnosed (when incident overweight/obesity was not considered). This is noteworthy given that a recent pooled analysis of 97 prospective cohort studies observed that the increase in risk of coronary heart disease and stroke with higher BMI was largely mediated by hypertension, which accounted for 31–65% of excess risk of these outcomes in that study.[25] Among those who had a BMI <25 kg/m^2^ at accession, incident overweight/obesity was the earliest appearing diagnosis, prior to hypertension, indicating a burgeoning problem in the population, one that typically leads to increased risk of other cardiometabolic conditions later in life.

In exploratory analyses, we observed that absent a CRF diagnosis, overweight and obese Soldiers at accession had shorter average follow-up times (i.e., service duration), likely indicating they left the Army earlier than their normal-weight counterparts. This observation is consistent with previous reports from Armed Forces health surveillance data (1988–2010), which observed a median 1.21-year shorter duration of service in Soldiers following a diagnosis of overweight/obesity versus matched controls.[18] Soldiers may be discharged if they are unable to lose weight (or regain weight following weight loss in Basic Combat Training) and/or are unable to comply with ongoing screening weight-for-height criteria and ultimately, body fat requirements.[18] Given that an incident overweight/obesity diagnosis may be a mediating factor and/or a source of follow-up bias in subsequent risk of another CRF, in secondary models we also adjusted for this diagnosis if it preceded the CRF of interest. Hazard ratios were attenuated, but substantively unchanged, suggesting either that these incident diagnoses of overweight/obesity were underreported or that excess adiposity earlier in adulthood has long-term effects on subsequent CRF risk that are not fully accounted for by overweight/obesity later in adulthood.

In addition, in those with an incident CRF, adjusted mean time to diagnoses (and mean age of diagnoses) was shorter for overweight/obese Soldiers than for normal-weight Soldiers, except for impaired glucose/insulin disorder, indicating earlier onset of most cardiometabolic risk factors in heavier Soldiers. Thus, the health burden and health care costs associated with cardiometabolic disease typically begin earlier in life for those with excess weight, even though observed differences were relatively small in the present population. Packnett, *et al*. observed that Soldiers with a BMI ≥25 kg/m^2^ had incrementally higher odds of all-cause and medical discharge during the first year of service, compared to those with a BMI of 24–24.9 kg/m^2^.[17] Indeed, the rigors of the first year likely do not impact all Soldiers in the same way. A study on the effects of Basic Combat Training on cardiometabolic risk factors in 209 new recruits observed that, at baseline, 22% of male and 4% of female recruits were obese, total cholesterol exceeded recommended levels in 8% of men and 9% of women, LDL cholesterol was high in 44% and 31%, respectively, triglycerides were high in 6% and 3%, respectively, and glucose was high in 11% and 4%, respectively. HDL cholesterol was below recommended levels in 29% of men and 38% of women.[14] After 9 weeks, 12% of men and 0% of women remained obese, 5% and 0%, respectively, had hyperglycemia, 20% and 21%, respectively, had high LDL, 31% and 49%, respectively, still had low HDL, and 1% and 4%, respectively, still had high triglycerides.[14] In the present study, although we noted a lower prevalence of overweight/obesity at accession in women which is consistent with Pasiakos, *et al*.[14] and likely due to stricter height and weight standards for women, excess body weight at accession raised risk of CRFs equivalently in both men and women.

Packnett and colleagues[17] previously observed a U-shaped pattern to risk of attrition: Soldiers with a BMI <17 kg/m^2^ were 35% and 45% more likely than those with a BMI of 24–24.9 kg/m^2^ to have an all-cause or medical discharge, respectively, which may be due cardiometabolic health or other causes (e.g., injury[36]). While underweight Soldiers had lower risk of hypertension, dyslipidemia, and overweight/obesity than normal-weight Soldiers in the present study, there were not significant differences in time to diagnoses (or age at diagnoses) between underweight and normal-weight Soldiers. This may be due to the relatively low number of underweight Soldiers and/or overall diagnoses observed in this subset of the population. However, we did observe a slight but significant earlier onset of glucose/insulin disorders in normal-weight and underweight Soldiers than for overweight or obese Soldiers, whether these disorders were broadly or strictly defined. Some have suggested that pathways of insulin resistance and T2D are at least partially independent of body weight, and our results may reflect such biological pathways.[37] At least one study in a civilian population has indicated that BMI in the context of other risk factors is not a significant predictor of age of onset of type 2 diabetes for onset between ages 18–29 years, instead observing a more dominant role of family history (and insulin) in this age group.[38] Nevertheless, in the present analysis, Soldiers who were overweight or obese at accession were ultimately at higher risk of glucose/insulin disorders, despite their approximately 3–8-month later diagnostic appearance.

The present analysis has limitations. First, we relied on diagnostic codes from inpatient and outpatient medical records as proxies for incident disorders. While our data sets include deployment medical data, deployment medical encounters are likely underrepresented in the data set. We did not have access to incident anthropometric or lab measurements, which would have allowed for confirmation of diagnoses. However, the use of diagnostic codes in large health systems for some conditions (e.g., diabetes), have been shown to have high sensitivity, specificity, and positive predictive value in outpatient and general internal medicine clinics.[39] On the other hand, the increasing frequency of overweight/obesity diagnoses in Soldiers between 1998 and 2010,[40] high self-reported prevalence of overweight/obesity (e.g., 61.8% in 2008[41]), and the presently observed prevalence of overweight/obesity at accession, all point to diagnoses of overweight/obesity likely not being reliably recorded by ICD codes, an observation supported by several reports showing poor sensitivity of ICD codes versus chart reviews, resulting in underestimated prevalence of these conditions when using ICD data alone.[42,43] Underreporting of overweight/obesity, in particular, may occur if clinical notes do not explicitly state overweight or obesity, the limited coding time may be devoted to other clinical conditions, and so on.[42,43] Because of this, at least one author group has suggested that overweight/obesity coding from weight and height should occur automatically within electronic health records based on measured weight and height.[16] In addition, the metabolic syndrome has been noted to be a rarely used diagnostic code,[44] a phenomenon also observed in our population. Finally, we lacked waist and hip circumference, body fat, and fitness data, all of which may have allowed us to further refine risk associated with excess body weight versus related factors.

Notably, our study includes a large sample size, inpatient and outpatient medical records, and long-term follow-up. The observations presented herein are largely consistent with prior research in US Army Soldiers, but add to our knowledge of the increasing risks associated with excess body weight even in this ostensibly young, physically active population. Our data indicate that overweight at accession increases, and obesity at accession at least doubles the risk of incident cardiometabolic risk factors, which may have important implications for weight-related accession standards and programming in Active Duty Soldiers, to minimize the burdens associated with excess body weight and subsequent disease. Finally, since cardiovascular fitness is an important mediator of the cardiometabolic risks associated with excess adiposity, future efforts should focus on differentiating the roles of fitness and adiposity, as well as related behavioral risk factors, on cardiometabolic health.

## Supporting Information

S1 Table Screening Table Weights Based on US Army Regulation 40–501. (PDF)

S2 Table Body Fat Percentage Standards Based on US Army Regulation 40–501. (PDF)

S3 Table ICD-9 Codes Used to Categorize Broadly- and Strictly-Defined Cardiometabolic Risk Factors and Behavioral Risk Factors. (PDF)

S4 Table Hazard Ratios (95% Confidence Intervals) of Strictly Defined Incident Cardiometabolic Risk Factors across Body Mass Index Categories at Accession among 731,014 US Army Entrants, 2001–2011. (PDF)

S5 Table Hazard Ratios (95% Confidence Intervals) of Broadly Defined Incident Cardiometabolic Risk Factors across Standard Table Weight Categories at Accession among 731,014 US Army Entrants, 2001–2011. (PDF)

## Abbreviations

BMI: body mass index
CAPER: Comprehensive Ambulatory Provider Encounter Record
CI: confidence interval
CRF: cardiometabolic risk factor
DoD: Department of Defense
FM: Field Manual
HR: hazard ratio
ICD: International Classification of Diseases
SADR: Standard Ambulatory Data Record
SIDR: Standard Inpatient Data Record
STW: Standard Table Weight
TED-I: TRICARE Encounter Data—Institutional
TED-NI: TRICARE Encounter Data—Non-Institutional
TAIHOD: Total Army Injury and Health Outcomes Database
US: United States
