# Supplementary material for: Body Mass Index at Accession and Incident Cardiometabolic Risk Factors in US Army Soldiers, 2001–2011
Source: PLoS One. 2017 Jan 17;12(1):e0170144. doi: 10.1371/journal.pone.0170144 (PMC5241140; doi:10.1371/journal.pone.0170144)
Supplement: S1 Table — (PDF) [file pone.0170144.s001.pdf]

Supporting Information

Hruby, *et al*. Body Mass Index at Accession and Incident Cardiometabolic Risk Factors in US Army Soldiers, 2001–2011.

S1 Table. Screening Table Weights Based on US Army Regulation 40-501.*

		**October 1, 1991 through January 13, 2008**					**January 14, 2008 forward**				
			**Maximum weight by age** (lbs)					**Maximum weight by age** (lbs)			
**Sex**	**Height** (in)	**Min. Weight** (lbs)	**17-20 yrs**	**21-27 yrs**	**28-39 yrs**	**≥40 yrs**	**Min. Weight** (lbs)	**17-20 yrs**	**21-27 yrs**	**28-39 yrs**	**≥40 yrs**
Male	60	100	139	141	143	146	**97**	139	141	143	146
	61	102	144	146	148	151	**100**	144	146	148	151
	62	103	148	150	153	156	**104**	148	150	153	156
	63	104	153	155	158	161	**107**	153	155	158	161
	64	105	158	160	163	166	**110**	158	160	163	166
	65	106	163	165	168	171	**114**	163	165	168	171
	66	107	168	170	173	177	**117**	168	170	173	177
	67	111	174	176	179	182	**121**	174	176	179	182
	68	115	179	181	184	187	**125**	179	181	184	187
	69	119	184	186	189	193	**128**	184	186	189	193
	70	123	189	192	195	199	**132**	189	192	195	199
	71	127	194	197	201	204	**136**	194	197	201	204
	72	131	200	203	206	210	**140**	200	203	206	210
	73	135	205	208	212	216	**144**	205	208	212	216
	74	139	211	214	218	222	**148**	211	214	218	222
	75	143	217	220	224	228	**152**	217	220	224	228
	76	147	223	226	230	234	**156**	223	226	230	234
	77	151	229	232	236	240	**160**	229	232	236	240
	78	153	235	238	242	247	**164**	235	238	242	247
	79	159	241	244	248	253	**168**	241	244	248	253
	80	166	247	250	255	259	**173**	247	250	255	259

Supporting Information

Hruby, *et al*. Body Mass Index at Accession and Incident Cardiometabolic Risk Factors in US Army Soldiers, 2001–2011.

		October 1, 1991 through January 13, 2008					January 14, 2008 forward				
			**Maximum weight by age** (lbs)					**Maximum weight by age** (lbs)			
**Sex**	**Height** (in)	**Min Weight** (lbs)	**17-20 yrs**	**21-27 yrs**	**28-39 yrs**	**≥40 yrs**	**Min Weight** (lbs)	**17-20 yrs**	**21-27 yrs**	**28-39 yrs**	**≥40 yrs**
Female	58	90	112	115	119	122	**91**	**122**	**124**	**126**	**127**
	59	92	116	119	123	126	**94**	**127**	**128**	**130**	**131**
	60	94	120	123	127	130	**97**	**132**	**134**	**135**	**136**
	61	96	124	127	131	135	**100**	**136**	**137**	**139**	**141**
	62	98	129	132	137	139	**104**	**140**	**141**	**144**	**145**
	63	100	133	137	141	144	**107**	**145**	**147**	**148**	**149**
	64	102	137	141	145	148	**110**	**149**	**151**	**153**	**154**
	65	104	141	145	149	153	**114**	**154**	**156**	**158**	**160**
	66	106	146	150	154	158	**117**	**160**	**160**	**162**	**165**
	67	109	149	154	159	162	**121**	**163**	**166**	**168**	**169**
	68	112	154	159	164	167	**125**	**168**	**171**	**173**	**174**
	69	115	158	163	168	172	**128**	**173**	**176**	**178**	**180**
	70	118	163	168	173	177	**132**	**178**	**181**	**183**	**185**
	71	122	167	172	177	182	**136**	**183**	**186**	**188**	**191**
	72	125	172	177	183	188	**140**	**189**	**191**	**194**	**196**
	73	128	177	182	188	193	**144**	**194**	**196**	**200**	**202**
	74	130	183	189	194	198	**148**	**199**	**203**	**204**	**206**
	75	133	188	194	200	204	**152**	**205**	**208**	**210**	**212**
	76	136	194	200	206	209	**156**	**210**	**213**	**215**	**216**
	77	139	199	205	211	215	**160**	**216**	**219**	**221**	**223**
	78	141	204	210	216	220	**164**	**222**	**224**	**227**	**229**
	79	144	209	215	222	226	**168**	**227**	**230**	**234**	**236**
	80	147	214	220	227	232	**173**	**233**	**236**	**240**	**241**

Bold text indicates changed body fat standard from prior interval.

*Adapted from US Department of the Army. Standards of Medical Fitness, Army Regulation 40-501. US Department of the Army, Washington, D.C.; Available at: http://armypubs.army.mil/epubs/40_Series_Collection_1.html. In brief, there were three primary intervals in the time period of interest: (1) October 1991

(introduction of body fat standards) through June 2006; (2) July 2006 (increase in body fat standards for 17–20-year olds effective July 2006 and following widespread introduction of the Assessment of Recruit Motivation and Strength Study (ARMS) fitness-based waivers in February 2006 [references below]) through December 2007; and (3) January 2008 (changes to minimum body weight in men and women, and maximum body weight in women) through December 2011 (study cutoff date). Results of ARMS appeared in: Niebuhr DW, Scott CT, Li Y, Bedno SA, Han W, Powers TE. Preaccession fitness and body composition as predictors of attrition in US Army recruits. *Mil Med*. 2009;174(7):695-701; and in Niebuhr DW, Page WF, Cowan DN, Urban N, Gubata ME, Richard P. Cost-effectiveness analysis of the US Army Assessment of Recruit Motivation and Strength (ARMS) program. *Mil Med*. 2013;178(10):1102-1110. doi:10.7205/MILMED-D-13-00108.
