# Supplementary material for: Body Mass Index at Accession and Incident Cardiometabolic Risk Factors in US Army Soldiers, 2001–2011
Source: PLoS One. 2017 Jan 17;12(1):e0170144. doi: 10.1371/journal.pone.0170144 (PMC5241140; doi:10.1371/journal.pone.0170144)
Supplement: S2 Table — (PDF) [file pone.0170144.s002.pdf]

Supporting Information

Hruby, *et al*. Body Mass Index at Accession and Incident Cardiometabolic Risk Factors in US Army Soldiers, 2001–2011.

S2 Table. Body Fat Percentage Standards Based on US Army Regulation 40-501.*

	Maximum body fat by age (years)			
**Valid Dates**	**17–20 years**	**21–27 years**	**28–39 years**	**≥40 years**
	**Male**			
January 1991 through June 2006	24%	26%	28%	30%
July 2006 through December 2011	**26%**	26%	28%	30%
	**Female**			
	**17–20 years**	**21–27 years**	**28–39 years**	**≥40 years**
January 1991 through June 2006	30%	32%	34%	36%
July 2006 through December 2011	**32%**	32%	34%	36%

Bold text indicates changed body fat standard from prior interval.

*Adapted from US Department of the Army. Standards of Medical Fitness, Army Regulation 40-501. US Department of the Army, Washington, D.C.; Available at: http://armypubs.army.mil/epubs/40_Series_Collection_1.html. In brief, there were three primary intervals in the time period of interest: (1) October 1991 (introduction of body fat standards) through June 2006; (2) July 2006 (increase in body fat standards for 17–20-year olds effective July 2006 and following widespread introduction of the Assessment of Recruit Motivation and Strength Study (ARMS) fitness-based waivers in February 2006 [references below]) through December 2007; and (3) January 2008 (changes to minimum body weight in men and women, and maximum body weight in women) through December 2011 (study cutoff date). Results of ARMS appeared in: Niebuhr DW, Scott CT, Li Y, Bedno SA, Han W, Powers TE. Preaccession fitness and body composition as predictors of attrition in US Army recruits. *Mil Med*. 2009;174(7):695-701; and in Niebuhr DW, Page WF, Cowan DN, Urban N, Gubata ME, Richard P. Cost-effectiveness analysis of the US Army Assessment of Recruit Motivation and Strength (ARMS) program. *Mil Med*. 2013;178(10):1102-1110. doi:10.7205/MILMED-D-13-00108.
