# Supplementary material for: Body Mass Index at Accession and Incident Cardiometabolic Risk Factors in US Army Soldiers, 2001–2011
Source: PLoS One. 2017 Jan 17;12(1):e0170144. doi: 10.1371/journal.pone.0170144 (PMC5241140; doi:10.1371/journal.pone.0170144)
Supplement: S3 Table — (PDF) [file pone.0170144.s003.pdf]

Supporting Information

Hruby, *et al*. Body Mass Index at Accession and Incident Cardiometabolic Risk Factors in US Army Soldiers, 2001–2011.

S3 Table. ICD-9 Codes Used to Categorize Broadly- and Strictly-Defined Cardiometabolic Risk Factors and Behavioral Risk Factors.

**Cardiometabolic Risk Factors**	**Strict Definition Codes**	**Broad Definition Codes**
Impaired glucose disorder (hyperglycemia / hyperinsulinemia / type 2 diabetes)	250.x0–250.x2 Diabetes mellitus<br>790.21 Impaired fasting glucose<br>790.22 Impaired glucose tolerance test	*Strict plus:*<br>249.xx Secondary diabetes mellitus<br>251.1 Hyperinsulinism<br>362.0–362.07 Diabetic retinopathy<br>365.44 Diabetic glaucoma<br>366.41 Diabetic cataract<br>790.2 Abnormal glucose<br>791.5 Glycosuria, nonspecific on urine examination<br>V58.67 Long-term current insulin use
Overweight or obesity	278.0 Overweight, obesity, and other hyperalimentation<br>278.00 Obesity, unspecified<br>278.01 Morbid obesity<br>278.02 Overweight<br>V85.2 BMI 25–29 kg/m^2)<br>V85.3 BMI 30–39 kg/m^2)<br>V85.4 BMI 40+ kg/m^2)	*Strict plus:*<br>783.6 Hyperphagia
Hypertension	401.x Essential hypertension	*Strict plus:*<br>402.x Hypertensive heart disease<br>403.x Hypertensive chronic kidney disease<br>404.x Hypertensive heart and chronic kidney disease<br>405.x Secondary hypertension<br>796.2 Elevated blood pressure without diagnosis of hypertension<br>997.91 Complications affecting specified body systems, not elsewhere classified: Hypertension
Dyslipidemia	272.1 Pure hyperglyceridemia<br>272.5x Lipoprotein deficiency	*Strict, plus:*<br>All 272 Lipid disorders
Metabolic syndrome	277.7 Dysmetabolic Syndrome X	*n/a*
**Behavioral Risk Factors**		*[used in additional models to assess potential confounding of BMI-cardiometabolic risk factor relationship]*
Smoking / tobacco / drug		292.0 Abstinence and/or withdrawal symptoms or syndrome<br>292.9 Drug-related disorder not otherwise specified<br>305.1 Tobacco use disorder
Depression		293.83 Mood disorder<br>296.xx Bipolar disorder<br>300.4 Dysthymic disorder<br>311 Depressive disorder
Anxiety		293.84 Anxiety disorder<br>300.0x Anxiety disorders [including generalized, panic, etc.]

Supporting Information

Hruby, *et al*. Body Mass Index at Accession and Incident Cardiometabolic Risk Factors in US Army Soldiers, 2001–2011.

Posttraumatic stress disorder		308.3 Acute stress reaction<br>309.81 Posttraumatic stress disorder
Alcohol		291.81 Alcohol withdrawal<br>291.9 Unspecified alcohol-induced mental disorders<br>303.0x Acute alcoholic intoxication in alcoholism<br>303.9x Alcohol dependence<br>305.0x Alcohol abuse
