# Supplementary material for: Body Mass Index at Accession and Incident Cardiometabolic Risk Factors in US Army Soldiers, 2001–2011
Source: PLoS One. 2017 Jan 17;12(1):e0170144. doi: 10.1371/journal.pone.0170144 (PMC5241140; doi:10.1371/journal.pone.0170144)
Supplement: S4 Table — (PDF) [file pone.0170144.s004.pdf]

Supporting Information

Hruby, *et al*. Body Mass Index at Accession and Incident Cardiometabolic Risk Factors in US Army Soldiers, 2001–2011.

S4 Table. Hazard Ratios (95% Confidence Intervals) of Strictly Defined Incident Cardiometabolic Risk Factors across Body Mass Index Categories at Accession among 731,014 US Army Entrants, 2001–2011.

			**Body Mass Index Category (kg/m^2)**			
**Outcome**	**Model***	**Total Events**	**Underweight** (<18.5)	**Normal weight** (18.5–<25)	**Overweight** (25–<30)	**Obese** (≥30)
Metabolic syndrome (single code)	*Events*	228	2	41	105	80
	*Follow-up Time***		690,411	16,038,189	9,970,038	2,782,406
	*Crude Rate***		0.0003	0.0003	0.001	0.003
	Model 1		1.10 (0.27–4.55)	1 *(ref.)*	4.19 (2.91–6.03)	13.51 (9.12–20.01)
	Model 2		1.10 (0.27–4.53)	1 *(ref.)*	4.13 (2.87–5.94)	13.36 (9.00–19.83)
Overweight/obesity†	*Events*	5298	50	5,248	--	--
	Follow-up Time		688,605	15,883,433	--	--
	Crude Rate		0.007	0.033	--	--
	Model 1		0.19 (0.15–0.25)	1 *(ref.)*	--	--
	Model 2		0.19 (0.15–0.26)	1 *(ref.)*	--	--
Impaired glucose/ insulin disorder	*Events*	2602	42	1,053	992	515
	Follow-up Time		689,236	16,015,179	9,953,222	2,775,452
	Crude Rate		0.006	0.007	0.010	0.019
	Model 1		0.89 (0.66–1.22)	1 *(ref.)*	1.52 (1.40–1.66)	3.32 (2.98–3.71)
	Model 2		0.89 (0.65–1.21)	1 *(ref.)*	1.51 (1.38–1.64)	3.20 (2.87–3.58)
Hypertension	*Events*	18192	167	6,417	7,738	3,870
	Follow-up Time		685,869	15,889,327	9,798,849	2,702,474
	Crude Rate		0.024	0.040	0.079	0.143
	Model 1		0.62 (0.54–0.73)	1 *(ref.)*	1.82 (1.76–1.88)	3.39 (3.26–3.53)
	Model 2		0.62 (0.54–0.73)	1 *(ref.)*	1.85 (1.79–1.92)	3.34 (3.21–3.48)

Supporting Information

Hruby, *et al*. Body Mass Index at Accession and Incident Cardiometabolic Risk Factors in US Army Soldiers, 2001–2011.

Dyslipidemia	*Events*	1638	9	423	750	456
	Follow-up Time		690,296	16,031,563	9,959,318	2,776,081
	Crude Rate		0.001	0.003	0.008	0.016
	Model 1		0.53 (0.27–1.02)	1 *(ref.)*	2.60 (2.30–2.93)	5.72 (5.00–6.54)
	Model 2		0.52 (0.27–1.01)	1 *(ref.)*	2.52 (2.23–2.84)	5.77 (5.04–6.61)

*Model adjustments as follows: Model 1 was adjusted for age at baseline (<20, 20–<30, 30–<40, 40+ years) and sex. Model 2 was adjusted as for Model 1, plus the following demographic covariates: race/ethnicity (white, black, Hispanic, Asian/Pacific Islander, Indian/Alaskan, other/unknown), educational attainment (<high school, some college/college, advanced degree, other/unknown), and marital status (never married, married, divorced/separated/widowed, other/unknown).

**Expressed as/in person-months.

†Among those with body mass index <25 kg/m^2 at baseline/accession, *N*=408,216.
