# Supplementary material for: Body Mass Index at Accession and Incident Cardiometabolic Risk Factors in US Army Soldiers, 2001–2011
Source: PLoS One. 2017 Jan 17;12(1):e0170144. doi: 10.1371/journal.pone.0170144 (PMC5241140; doi:10.1371/journal.pone.0170144)
Supplement: S5 Table — (PDF) [file pone.0170144.s005.pdf]

Supporting Information

Hruby, *et al*. Body Mass Index at Accession and Incident Cardiometabolic Risk Factors in US Army Soldiers, 2001–2011.

S5 Table. Hazard Ratios (95% Confidence Intervals) of Broadly Defined Incident Cardiometabolic Risk Factors across Standard Table Weight Categories at Accession among 731,014 US Army Entrants, 2001–2011.

			**Standard Table Weight Category**		
**Outcome**	**Model***	**Total Events**	**Under Criteria**	**Meet Criteria**	**Exceed Criteria**
Metabolic syndrome (single code)	*Events*	228	0	71	157
	*Follow-up Time***		233,892	21,229,021	8,018,132
	*Crude Rate***		0.000	0.0003	0.0020
	Model 1		--	1 *(ref.)*	5.84 (4.40–7.75)
	Model 2		--	1 *(ref.)*	5.72 (4.30–7.59)
Overweight/obesity†	*Events*	5,361	19	4,210	1,132
	Follow-up Time		233,529	15,895,587	441,842
	Crude Rate		0.008	0.026	0.256
	Model 1		0.30 (0.19–0.47)	1 *(ref.)*	3.83 (3.55–4.14)
	Model 2		0.30 (0.19–0.47)	1 *(ref.)*	3.78 (3.50–4.08)
Impaired glucose/insulin disorder	*Events*	3,880	28	2,206	1,646
	Follow-up Time		233,391	21,182,952	7,991,029
	Crude Rate		0.012	0.010	0.021
	Model 1		1.28 (0.88–1.86)	1 *(ref.)*	1.81 (1.70–1.94)
	Model 2		1.28 (0.88–1.87)	1 *(ref.)*	1.79 (1.68–1.91)
Hypertension	*Events*	2,602	71	14,579	11,723
	Follow-up Time		232,667	20,879,443	7,756,939
	Crude Rate		0.031	0.070	0.151
	Model 1		0.54 (0.43–0.69)	1 *(ref.)*	2.19 (2.13–2.24)
	Model 2		0.54 (0.43–0.68)	1 *(ref.)*	2.18 (2.13–2.24)

Dyslipidemia	*Events*	13,404	31	7,500	5,873
	Follow-up Time		233,481	21,068,528	7,899,345
	Crude Rate		0.013	0.036	0.074
	Model 1		0.56 (0.40–0.80)	1 *(ref.)*	2.16 (2.08–2.23)
	Model 2		0.57 (0.40–0.81)	1 *(ref.)*	2.12 (2.05–2.20)

*Model adjustments as follows: Model 1 was adjusted for age at baseline (<20, 20–<30, 30–<40, 40+ years) and sex. Model 2 was adjusted as for Model 1, plus the following demographic covariates: race/ethnicity (white, black, Hispanic, Asian/Pacific Islander, Indian/Alaskan, other/unknown), educational attainment (<high school, some college/college, advanced degree, other/unknown), and marital status (never married, married, divorced/separated/widowed, other/unknown). Standard Table Weights are adapted from US Department of the Army. Standards of Medical Fitness, Army Regulation 40-501. US Department of the Army, Washington, D.C.; Available at: http://armypubs.army.mil/epubs/40_Series_Collection_1.html.
**Expressed as/in person-months.
†Among those with body mass index <25 kg/m^2 at baseline/accession, *N*=408,216.
